# Owner and Veterinarian Perceptions About Use of a Canine Quality of Life Survey in Primary Care Settings

**DOI:** 10.3389/fvets.2020.00089

**Published:** 2020-02-26

**Authors:** Kennedy K. Mwacalimba, Francesca M. Contadini, Nathaniel Spofford, Karen Lopez, Aimee Hunt, Andrea Wright, Elizabeth M. Lund, Larissa Minicucci

**Affiliations:** ^1^Outcomes Research, Zoetis Petcare, Parsippany, NJ, United States; ^2^Department of Veterinary Epidemiology and Public Health, University of Surrey, Guildford, United Kingdom; ^3^Veterinary Analytics, Banfield Pet Hospital, Vancouver, WA, United States; ^4^Delaware Department of Agriculture, Dover, DE, United States; ^5^Veterinary Public Health, University of Minnesota College of Veterinary Medicine, St. Paul, MN, United States; ^6^Veterinary Informatics, Compassion-First Pet Hospitals, Vancouver, WA, United States

**Keywords:** preventive care, client communication, veterinary staff, canine, health related quality of life

## Abstract

This paper describes dog owner and veterinarian perceptions around the use of a validated canine quality of life (QOL) survey to facilitate wellness conversations in two clinical settings: a veterinary teaching hospital (pilot, Phase 1) and five corporate general practice hospitals (Phase 2). Phase 1 results showed that dog owners felt the survey was valuable for understanding their dog's QOL, with 81% of owners expressing interest in learning more about canine QOL. Phase 2 reinforced owner perceptions about the survey conveyed during the pilot phase, and veterinarians reported that the survey facilitated client communication related to preventive care without increasing consultation time. These results demonstrate that beyond using QOL assessments to track patient health, the use of a QOL survey during veterinary visits could improve owner-veterinarian discussions around QOL, wellness, services and preventive care. To fully realize these benefits in clinical settings, veterinary staff preparation may be needed to communicate the purpose of QOL assessments to clients and thus facilitate deeper conversations about client needs and concerns. Key tools for achieving these could therefore include (1) sufficient veterinary team training to understand the QOL assessment and its purpose (2) training in how to communicate QOL to clients, and (3) reflexive use of QOL assessment results to engage clients in preventive care discussions. The veterinarian and client can then discuss the pros and cons of the various aspects of QOL and preventive care to arrive at a cooperative decision.

## Introduction

Quality of life (QOL) is a subjective interpretation of individual wellbeing, assessing interacting intrinsic and extrinsic factors that impact upon a single subject (1). The self-report is the gold standard for QOL in humans, but certain circumstances require assessments be made by an observer familiar with the individual (2). Pets cannot adequately communicate most aspects of QOL and must rely on an observer to report them. In most instances, the observer best placed to do this is the pet owner. The American Veterinary Medical Association U.S. Pet Ownership and Demographics Sourcebook states that in 2011, over 63% of owners saw their pets as family members (3). Although consistent across the age spectrum, this representation is most notable with Millennials, where the “humanization of pets” continues to be a driving factor (4). Furthermore, advancements in veterinary medical knowledge and technology have contributed to longer pet lifespans (5), allowing owners to enjoy more time with their animal companions while increasing the importance of having regular conversations about their pet's QOL (6–9).

Canine QOL surveys account for a dog's physical health, as well as “needs satisfaction, sense of control, social relationships, the extent of physical or emotional discomfort, and management of stress” (8). They highlight the importance of preventive care in maintaining health and wellbeing (10–12). Using QOL surveys in routine clinical practice may therefore also facilitate better communication between veterinarians and pet owners by increasing the depth and effectiveness of preventive care and wellbeing conversations, and thus increase overall satisfaction with veterinary care.

**Figure 1:**
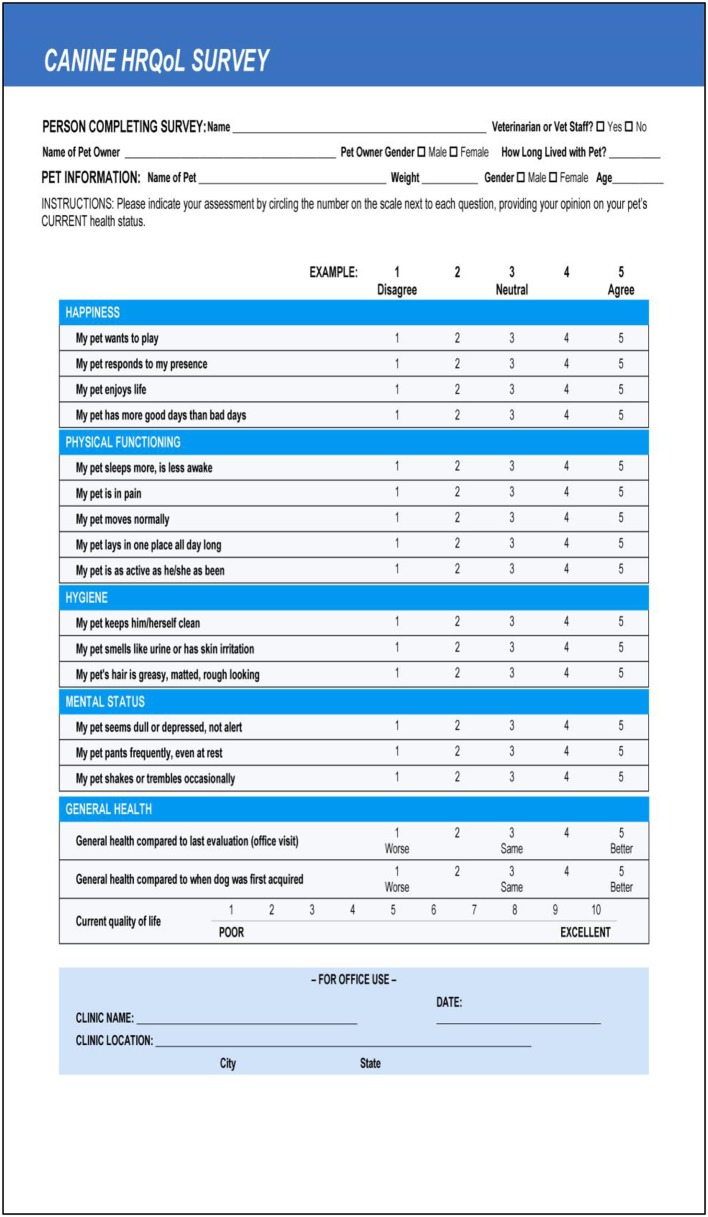
The Healthy Dog QOL Survey.

In 2013, a Canine Health Related QOL (HRQOL) survey was developed and validated for the long-term evaluation of QOL in healthy dogs (8). The basic premise of this tool is that QOL decreases as part of the natural process of healthy aging. This can be captured in a repeated HRQOL measure. The resulting evaluations could then be used to guide discussions between pet owners and veterinarians on long term care (8). The first 15 questions of this survey use a Likert scale to rank the pet owner's perception of their pet's status in the areas of happiness, physical functioning, hygiene, and mental condition. Two questions focus on the patient's current health in relation to the previous office visit, as well as when the pet was initially acquired (1–5 scale; 1 = worse, 3 = same, 5 = better). The final question asks the owner to rank the pet's current HRQOL on a scale of 1–10 (Direct HRQOL score), with 1 being poor and 10 being excellent. A proprietary algorithm is applied to obtain a calculated QOL score (Calculated HRQOL score), from responses to the first 15 questions (range 1–10) (Figure 1) (8).

To determine the effectiveness of this tool in facilitating wellness discussions between veterinarians and clients in routine practice, a two-year study was undertaken. Phase 1 was a pilot study to evaluate the usability of the Canine HRQOL survey in a single primary care facility. Phase 2 of the project evaluated the effectiveness of this survey to support clinical conversations in five corporate general practice veterinary hospitals. Phase 2 also obtained feedback from practitioners on how the HRQOL survey impacted client satisfaction and their perception of preventive care. In both phases, the Canine HRQOL survey was completed by pet owners at one timepoint.

## Materials and Methods

Surveys and study design were reviewed by the University of Minnesota's Institutional Review Board and determined to be exempt from review. All participants (i.e., pet owners) in both phases received a study overview, had to provide written consent prior to participation, and were incentivized with a $10 credit to their veterinary account for their participate in the study. Phase 1 of the project used a 16-item questionnaire to evaluate the ease of use of the Canine HRQOL tool by clients. It had 12 questions on the length of time needed to complete the HRQOL survey, appropriateness of length, difficulty of individual questions, interest in receiving additional information about canine HRQOL, and the nature of the client's relationship with their pet. Four questions covered pet owner demographics and patient signalment.

Phase 1 was conducted between October 19, 2015 and January 29, 2016, at the University of Minnesota Veterinary Medical Center (VMC) Primary Care Service. Clients were selected to participate if they were the owner of a canine patient being presented for a routine or sick visit. Clients were excluded from participation if they had completed the Canine HRQOL survey during a previous visit or were presenting a dog for euthanasia or terminal illness care. Participants were asked to complete the Canine HRQOL survey as well as the usability assessment. Hospital staff were provided written materials detailing the study requirements and enrollment criteria. These were coupled with in-person training by the research team.

**Figure 2:**
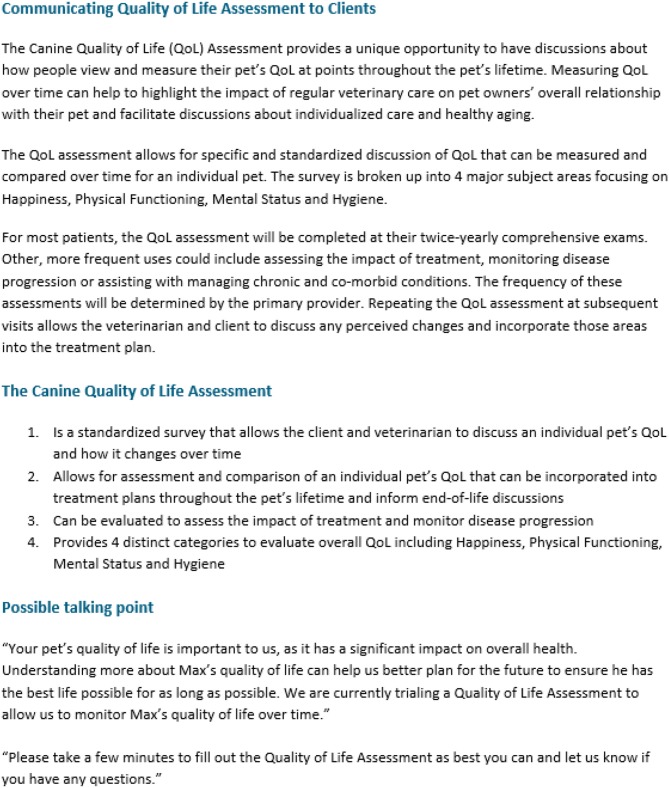
One-page guidance document.

Phase 2 was conducted between June 20, 2016 and September 30, 2016 at five general practice hospitals in the Minneapolis–Saint Paul metropolitan area. Inclusion criteria were the same as in Phase 1. Training for Phase 2 included the same components as Phase 1, supplemented with a webinar to coach hospital staff on administration of the Canine HRQOL survey. They were also provided with a one-page guidance document on how to communicate QOL assessments to clients. The guidance document explained the purpose of the HRQOL assessment and provided talking points for introducing and explaining the tool to clients (Figure 2). One of the hospitals initially selected requested removal from the trial due to staff turnover, which made it difficult for them to participate while maintaining normal business operations. A replacement hospital was enrolled from the same area.

**Figure 3:**
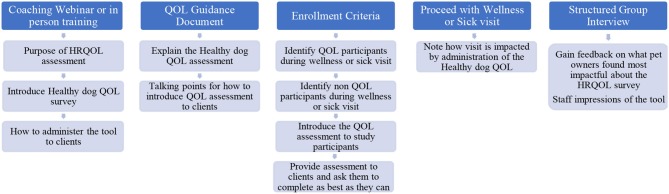
Hospital staff training process diagram.

Hospital clients were enrolled at the time of their visit into a group that was asked to complete the HRQOL survey prior to their consultation and a group of participants that completed their visit without the HRQOL assessment. An online 16-item questionnaire, adapted from Phase 1, was emailed to participants in both groups within one week of their enrollment visit. Furthermore, upon completion of Phase 2, a structured group interview was conducted with medical and management staff from each hospital location to gain feedback on what they perceived pet owners found most impactful about the HRQOL survey, as well as staff impressions of the tool (Figure 3).

Data from the usability assessments were entered into a Microsoft Access database by research team members. Data from the Canine HRQOL survey were entered into Qualtrics™ by VMC intake staff and transferred to Zoetis staff, who provided both a Direct and Calculated HRQOL score for each dog to University of Minnesota researchers.

### Data Analysis

Data were analyzed using Epi-Info™, Microsoft Excel and/or SAS®. Frequency tables were generated for all variables. Comparisons and results were based on proportion analysis with the SAS ProbNorm function (SAS 9.4, Cary, NC), using a two-sided test, at the 5% level of significance (*p* < 0.05). No statistical correction for multiple testing was performed. The Canine HRQOL survey was completed by pet owners at only one timepoint, and since the tool was developed for longitudinal use, QOL scores were summarized but not statistically analyzed. Qualitative feedback from pet owners and responses from focus group discussions with hospital staff were transcribed and reviewed for overarching themes. Drawing on the constant comparison techniques of grounded theory to classify the textual data (13), analysis of this qualitative data involved reading and re-reading the transcripts and identifying overarching themes by iteratively comparing responses and project objectives.

## Results

### Phase 1: Usability Assessment

One-hundred and fifty-one Canine HRQOL surveys were completed during Phase 1. Four of the corresponding usability assessments were returned blank and 17 were incomplete, leaving a total of 130 (86%) completed usability assessments for analysis.

The usability assessment results showed that the Canine HRQOL survey took less than 10 minutes to fill-out, with most respondents considering it easy to very easy to complete. Only 2% of respondents needed assistance to complete the survey. Most pet owners considered their dog to be a family member, companion or best friend. Only 1 respondent described their dog as a working dog. Additional respondent comments were variable but focused more on how the tool could be improved or its scope expanded. This suggested there was need to explain the purpose of the tool more clearly to staff and clients in the next phase (Table 1). Overall, respondents found the tool valuable and were interested in learning more about canine QOL, suggesting that the tool was usable in clinical settings (Table 2).

**Table 1:** Phase 1 Client feedback and interpretation on Canine QOL assessment.

**Quote**	**Interpretation**
*I don't know what the goal is, so it's hard to say… but maybe more questions around diet & exercise*	HRQOL context of use not defined; **Purpose of tool not clearly explained**
*My dog currently has cancer and there is no accommodation for a dog with a chronic illness*.	HRQOL context of use not defined, tool is validated for healthy dogs
*Would be nice to have a place to write comments*	HRQOL interpretation not defined
*questions 7-8-10-11-12 yes or no would be sufficient*	HRQOL context of use not defined; **Purpose of tool not clearly explained**
*I really like the idea of a Quality of Life assessment - felt like it could have been a bit longer if anything - maybe leave some space for written comments. Thanks!*	**Purpose of tool not clearly explained**
*I think her life is grand, but she may have a different opinion*	Define whose perspective is being captured in the assessment; **Purpose of tool not clearly explained**
*6 month old puppy, so not too much applied*	**Purpose of tool not clearly explained**
*Could be slightly longer; question on anxiety, actual playtime, exercise time, interactions with others*	**Purpose of tool not clearly explained**
*Time taken to complete survey unknown because broken up because of pet distraction*	Barrier to completion
*Perhaps modify those questions to distinguish between personality traits (trembling & sleeping) and mental/neuro & health issues*.	HRQOL context of use not defined, **Purpose of tool not clearly explained**
*I feel as though most people would already know the answer to their pet's quality of life before (without) taking the survey. Or at least I would hope so*.	**Purpose of tool not clearly explained**
*Feel this may be hard to do, very dependent on reason of visit*	**Purpose of tool not clearly explained**
*The paperwork seemed overwhelming and almost deterred me from completing. Once competed it did not take as long*	Barrier to completion
*I think it is great to assess and consider quality of life as part of canine health and wellness*	Perceived value
*Comparative questions spanning the dog's life are difficult to consider w/o context when I've had him since 8 weeks old*	HRQOL context of use not defined
*I like this idea and this research- its promise*	Perceived value

**Table 2:** Phase 1 usability assessment survey results.

**How easy or difficult was it for you to complete the Canine Quality of Life assessment?** 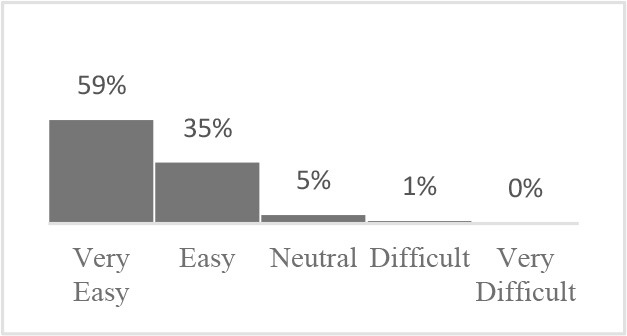	**Approximately how long did it take for you to complete the Canine Quality of Life assessment?** 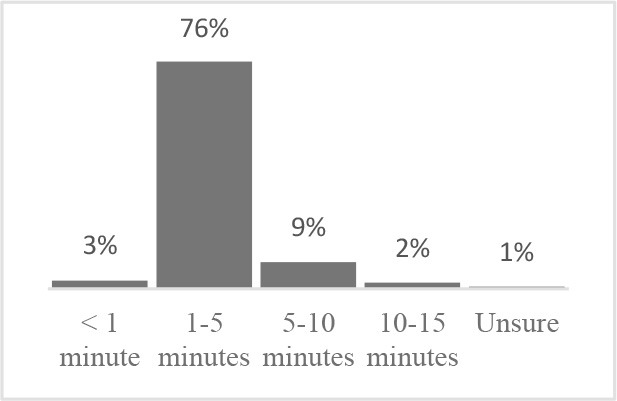	**How long was the Canine Quality of Life assessment?** 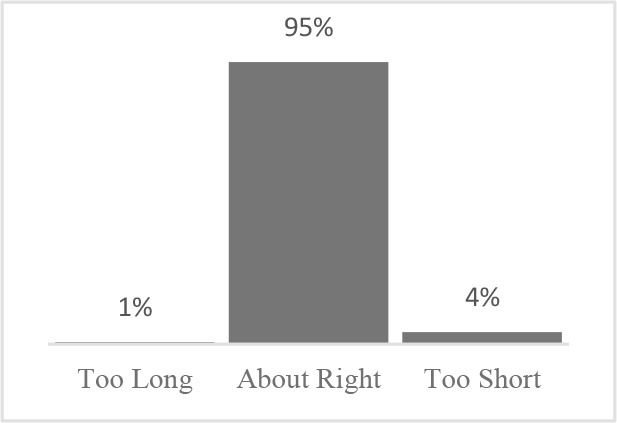	**Were the Canine Quality of Life assessment questions difficult to understand?** 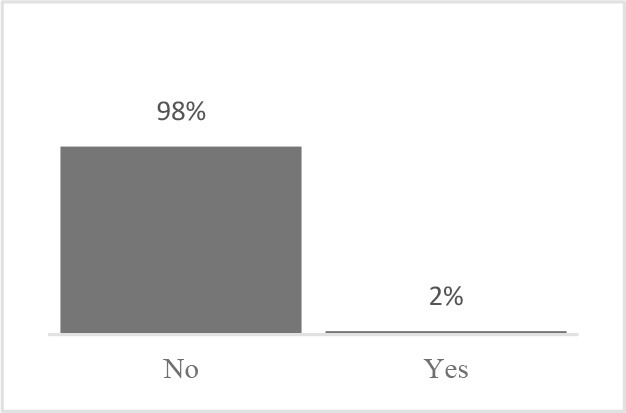
**Did you need help to complete the Canine Quality of Life assessment?** 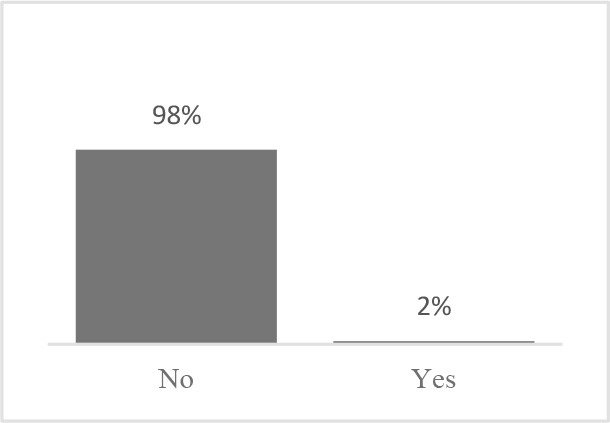	**Have you completed a Quality of Life assessment for your dog before?** 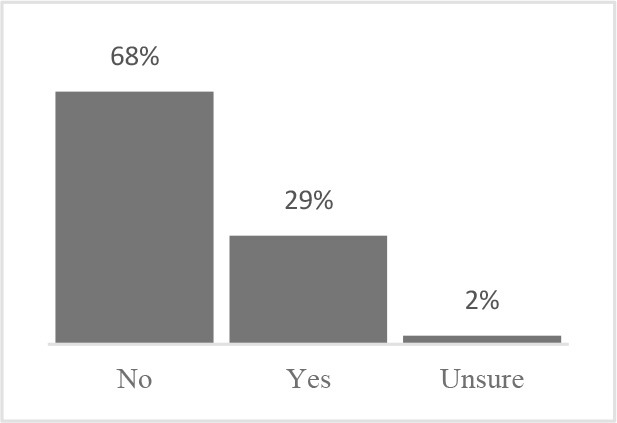	**How old is your dog?** 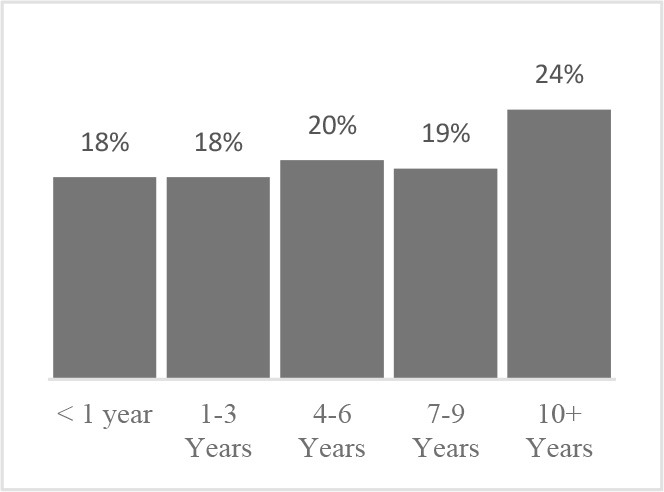	**How many years have you owned your dog?** 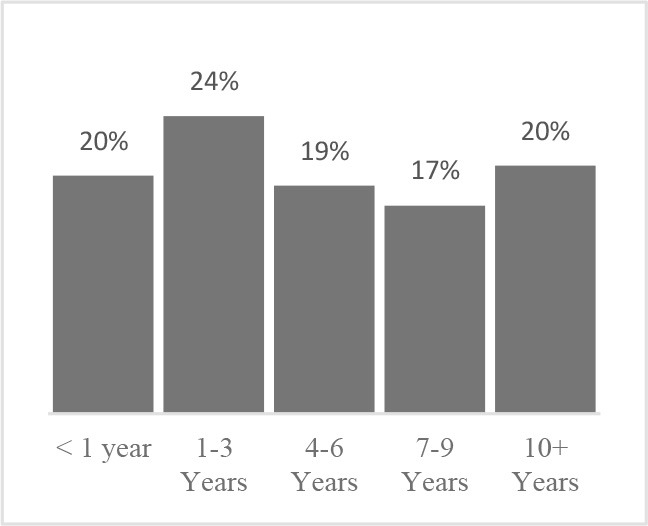
**What word best describes your relationship with your pet?** 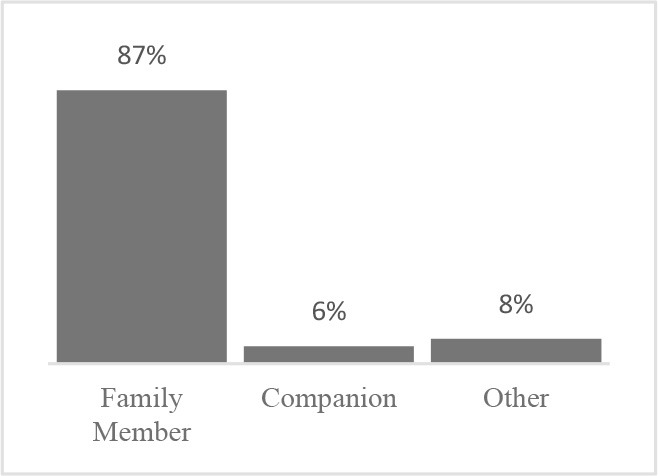	**How interested would you be in learning more about the Quality of Life of your dog?** 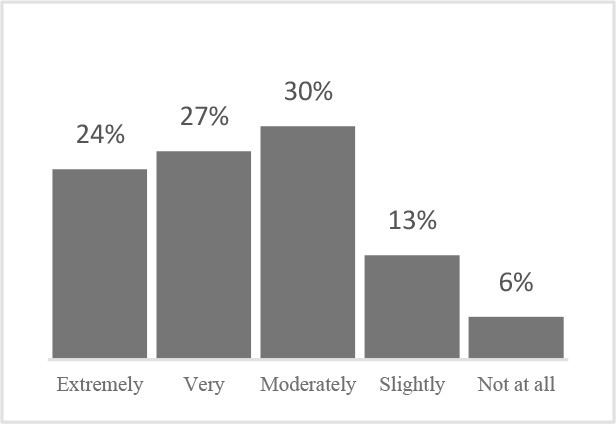		

### Phase 2: Veterinarian Perception and Client Satisfaction

One-hundred and ninety-one participants were enrolled in the study, 97 in the HRQOL group and 94 in the non-HRQOL group. Out of the enrolled total, 94 participants (49%) responded entirely to the online 16-item follow-up client satisfaction evaluation, 47 in the HRQOL group and 47 in the non-HRQOL group, giving the power to detect a minimum of 6% difference (alpha 0.05, power 0.80).

The two groups did not differ significantly in education level, age of pet or health status of pet as determined by the attending veterinarian. Eight baseline questions were used to compare client satisfaction responses between the HRQOL participants and non-HRQOL participants (Table 3). Both groups were satisfied to highly satisfied with the assessment visit. Participants in the non-HRQOL group (66%; *n* = 25) had a greater likelihood of reporting interest in learning more about their dog's HRQOL compared to the HRQOL group (53%; *n* = 31, Table 3). When evaluating the impact of the HRQOL survey on the veterinary visit experience, clients that completed the survey reported a more positive experience with regard to how adequately the veterinary teams addressed their questions related to preventive care (*p* = 0.0216) and other services (*p* = 0.0495), as well as the consideration that the team had toward the QOL of their pet (*p* = 0.0074), compared to the non-HRQOL group (Table 3). No other significant differences were noted between the two groups.

**Table 3:** Phase 2 Visit assessment comparison HRQOL vs. non HRQOL groups.

**Question**	**Response**	**HRQOL**	**NON-HRQOL**	***P*-Value**
Overall, how satisfied were you with your dog's most recent visit to XXX?	Satisfied	95.74%	95.74%	1.000
How interested would you be in learning more about your dog's quality of life?	Interested	53.19%	65.96%	0.2073
How interested would you be in discussing your dog's quality of life during your dog's routing checkup?	Interested	51.06%	68.09%	0.0927
The veterinary team took my dog's quality of life into consideration.	Agree	97.87%	80.85%	0.0074^*^
The veterinary team took my viewpoint about my dog's quality of life into consideration.	Agree	87.23%	82.98%	0.5623
The veterinary team answered all of my questions about my dog's routine checkup/preventive care.	Agree	100.00%	89.36%	0.0216^*^
The veterinary team answered all of my questions about the care and services my dog received.	Agree	97.87%	87.23%	0.0495^*^
I felt like I was given the opportunity to be involved in my dog's care.	Agree	95.74%	89.36%	0.2385

* *Indicates statistical significance*.

Similar to Phase 1, Phase 2 respondents that completed the HRQOL assessment considered the tool valuable, easy to complete, and expressed interest in learning more about QOL (Table 4). Although still perceived positively, respondents scored interest in completing the Canine QOL assessment before every routine checkup lower than other 5-point scale questions (Table 4).

**Table 4:** Phase 2 HRQOL usability assessment results.

**Question**	**Response**
Overall, how valuable is the Canine Quality of Life assessment to you as a pet owner?	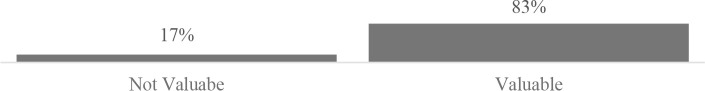
Approximately how long did it take for you to complete the Canine Quality of Life assessment?	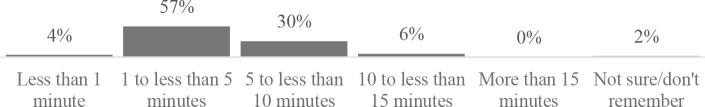
How easy or difficult was it for you to complete the Canine Quality of Life assessment?	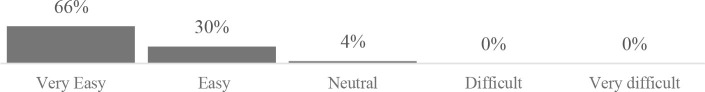
Did anyone help you complete the Canine Quality of Life assessment?	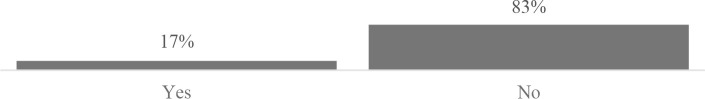
Which of the following statements best describes the length of the Canine Quality of Life assessment?	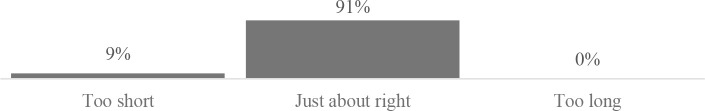
Did the Canine Quality of Life assessment cover all areas you feel are important to your dog's health-related quality of life?	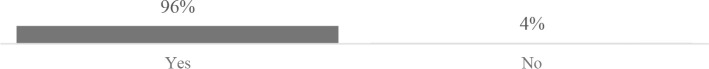
How interested would you be in completing the Canine Quality of Life assessment before every routine checkup?	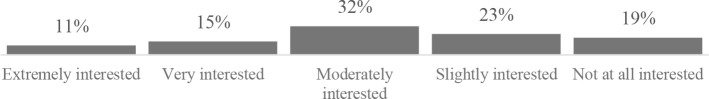

Qualitative feedback from the participating veterinary teams revealed the following key themes; (1) Staff generally had no difficulty in communicating QOL with clients, (2) the brevity of the HRQOL assessment did not add to time of consultation, (3) using the QOL tool stimulated discussion and helped elucidate the connection between services offered and Pet QOL and (4) the QOL tool increased the depth of discussion between veterinary staff and clients (Table 5). Therefore, the value of the canine HRQOL assessment in veterinary clinical practice was that it facilitated better communication between veterinarians and pet owners by increasing the depth and effectiveness of preventive care and wellbeing conversations.

**Table 5:** Summary of key themes from qualitative interviews with veterinary staff from participating hospitals.

**Theme**	**Summary response**
Easy to communicate QOL	•*Very comfortable discussing with clients* •*Very comfortable, no issue discussing; hadn't thought about discussing QOL with puppy/kitten and have started doing so sometimes*
Did not impact on time of consultation	•*Didn't really add any time before or after; It gave clients something to do and let the hospitals have a couple of extra minutes to catch up if running behind* •*Did not add much time, just a couple of minutes* •*No, it was complete by the time the doctor entered the room; the survey actually helped with visit because it kept client occupied for a few minutes while staff were finishing up*
Elucidated the connection between services offered and Pet QOL	•*Led to more conversations about how to fix underlying condition vs. keeping pet comfortable* •*Made it easier to talk about preventive care and clients may have seen increased benefit of Wellness Plans*
Increased depth of discussion	•*Helped to make it more of a team approach rather than just veterinarian (clients felt more a part of conversation)* •*Made it easier for families to talk about concerns at home rather than having doctors fishing for information; got clients thinking prior to appointment* •*The survey also got clients thinking about the appointment* •*Stimulated conversation and opened the door for further discussion*

### QOL Scores

In Phase 1, the mean Direct QOL score was 8.8 ± 1.3, with a median of 9 (range 4–10) and a mode of 10. The mean Calculated QOL score was 8.8 ± 0.8 with a median of 8.9 (range 5.7–10) and a mode of 9.4.

Results for Phase 2 showed a mean Direct QOL score of 8.9 ± 1.3, with a median of 9.5 (range 5–10) and a mode of 10. The calculated HRQOL score mean value was 8.5 ± 0.9, with a median of 8.8 (range: 5.1–9.6), and a mode of 9.2.

## Discussion

This paper described dog owner and veterinarian perceptions around the use of a canine QOL survey to facilitate wellness conversations in two clinical settings. Most respondents in this study described their pets as a companion, family member or best friend, highlighting the importance of the human-animal bond. The Human-Animal bond influences the care pets receive and increases the likelihood of clients seeking preventive care and accepting veterinarian health-care recommendations (14). Veterinary teams therefore need to respect the emotional bond between a client and their pet, while simultaneously communicating the medical realities of a pet's health to pet owners (15).

QOL assessments have been highlighted to be useful tools in discussions with veterinarians and clients (15). When the Canine HRQOL survey was implemented in our study, usability and satisfaction were consistently positive across phases, with clients expressing high levels of interest (>80%) in learning more about canine QOL. The pet owner and veterinary team can use QOL assessments to track the progression of the animal's condition (8). QOL tools vary in the circumstances they address, and veterinarians should identify and become familiar with assessments that are appropriate for the context (15). For instance, the Healthy Dog HRQOL tool is intended to track QOL overtime. Furthermore, pet owners' direct assessment of their dog's QOL is often higher than the calculated QoL score, as shown in this study. The calculated QoL score is linear, compared to the direct score. The dogs in our study needed a follow up assessment to determine the clinical significance of a change in scores. Follow up assessments were beyond the scope of this study. One challenge that the research team faced about midway through Phase 1 was handling inconsistency among members of intake staff regarding which clients should be asked to participate in the pilot study. Furthermore, the need to provide a clear context of use was noted in the qualitative feedback provided by pet owners in Phase 1 (Table 1).

To obviate the issues of client selection and client communication identified in Phase 1, more defined guidelines on implementing the tool were provided to the general practices enrolled in Phase 2. The development of a structured process for staff preparation in Phase 2, which included guidance on the context of use of the HRQOL assessment (Figure 2), helped to better define the tool's value for both veterinary staff and clients. Staff perceived that active use of the tool positively engaged clients' interest in preventive care by helping to elucidate the connection between services offered and Pet QOL (Table 5). Pet owners also reported positive experiences in several areas related to client communication. When compared to clients who did not receive the HRQOL survey, clients who completed the survey were more likely to report a better overall service, demonstrated by veterinary teams addressing their questions related to preventive and other services and care, as well as consideration of the QOL of their pet. This interaction could therefore be interpreted to be less transactional and more cooperative, and it has been suggested that a collaborative approach results in higher rates of client compliance with treatment plans and the highest levels of client satisfaction (16).

In this study, 66% of participants in the non-HRQOL group and 53% of participants in the HRQOL group reported interest in learning more about their dog's HRQOL (Table 3). Qualitatively, this trend may suggest a need for more discussion of QOL in clinical settings on the part of pet owners. Furthermore, although respondents scored interest in completing the Canine QOL assessment before every routine checkup positively, this was rated lower than other 5-point scale questions (Table 4). This could suggest a need to consider how often to complete the HRQOL assessment in clinical practice. Overall, clients who completed the HRQOL survey were more likely to report receiving a better overall service.

**Figure 4:**
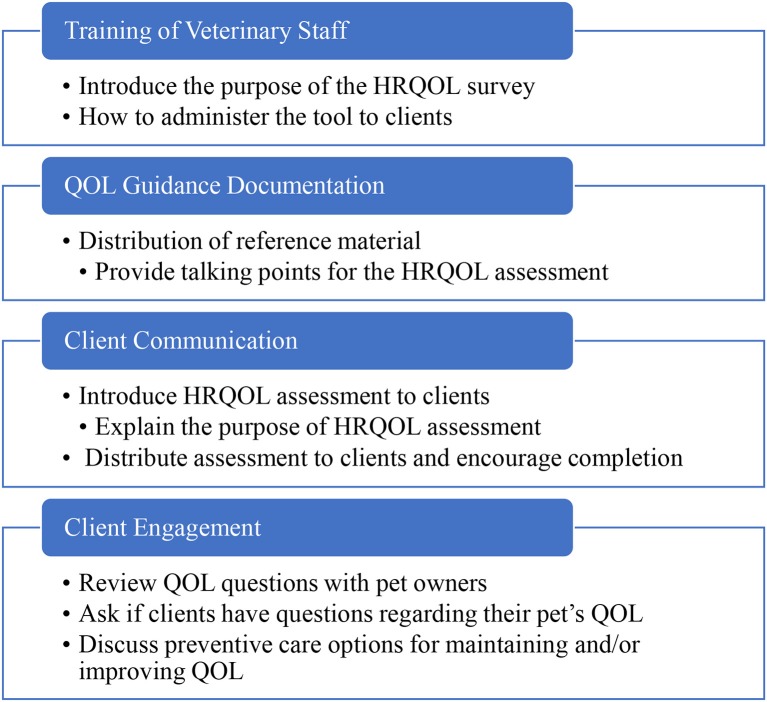
Conceptual framework for implementing a QOL assessment in clinical settings.

In the current study, a structured approach to implementing the HRQOL assessment elaborated the proximal benefits of using this tool, i.e., increased depth and effectiveness of conversations between veterinary teams and their clients and increased overall satisfaction with veterinary care. Key milestones for achieving these endpoints with similar QOL assessments could therefore include (1) sufficient training to understand the QOL assessment and its purpose (2) training in how to communicate QOL to clients, and (3) use of QOL assessments to engage clients in discussions of the importance of preventive care (Figure 4).

In both phases, missing or incomplete data impacted the study, especially during Phase 2 where only 49% of participants completed the follow up survey. Participants who felt more strongly about QOL may have been more willing to respond and thus report a higher interest in the topic and more favorable view of its impact than participants who were lost to follow-up. Additionally, one of the Phase 2 hospitals dropped out of the study and was replaced with another hospital, which may have skewed the positive perception reported by the remaining hospitals and led to a failure to detect implementation challenges that may occur in some settings. Finally, Phase 1 and Phase 2 were conducted in different veterinary contexts, so while Phase 1 results were used to develop tools for Phase 2, the results may not necessarily be repeatable in settings that do not have an ethos that supports client engagement on client satisfaction or improvement in client communication.

## Conclusion

Using the Canine HRQOL survey appeared to improve the flow of discussion during the veterinary visit and provided clinicians with an opportunity to address issues or concerns about the patient's QOL. This improved the client experience. Overall, the HRQOL survey appeared to be a highly usable tool that was well received by pet owners and added value to the veterinary visit for both clients and veterinarians. A structured process for veterinary staff training in implementing and communicating QOL to pet owners may better define the value of QOL assessments for both staff and clients during wellness visits.

## Data Availability Statement

The datasets generated for this study are available on request to the corresponding author.

## Ethics Statement

The studies involving human participants were reviewed and approved by University of Minnesota Institutional Review Board. The patients/participants provided their written informed consent to participate in this study.

## Author Contributions

KM, AW, EL, and LM: study design, data analysis, and manuscript development. FC and NS: study design, data collection, data analysis, and manuscript development. KL and AH study design, data collection, and data analysis.

### Conflict of Interest

KM and AW work for Zoetis Petcare that developed the Canine Health Related Quality of Life questionnaire. The remaining authors declare that the research was conducted in the absence of any commercial or financial relationships that could be construed as a potential conflict of interest.
